# Solar photo-Fenton optimization at neutral pH for microcontaminant removal at pilot plant scale

**DOI:** 10.1007/s11356-023-28988-7

**Published:** 2023-08-11

**Authors:** Mercedes Hinojosa, Isabel Oller, José María Quiroga, Sixto Malato, Agata Egea-Corbacho, Asunción Acevedo-Merino

**Affiliations:** 1 Department of Environmental Technologies, Faculty of Marine and Environmental Sciences, University of Cadiz, 11510, Puerto Real, Cádiz, Spain; 2 Plataforma Solar de Almería-CIEMAT, Carretera de Senés, Km 4.5, 04200 Tabernas, Almería Spain; 3 CIESOL, Joint Centre of the University of Almería-CIEMAT, 04120 Almería, Spain

**Keywords:** Antibiotics, Contaminants of emerging concern, Tertiary treatment, Fe-EDDS, Optimization, Solar photo-Fenton

## Abstract

The increasing occurrence of micropollutants in natural water bodies has medium to long-term effects on both aquatic life and human health. The aim of this study is to optimize the degradation of two pharmaceutical pollutants of emerging concern: amoxicillin and acetaminophen in aqueous solution at laboratory and pilot scale, by solar photo-Fenton process carried out at neutral pH using ethylenediamine-N,N′-disuccinic acid (EDDS) as a complexing agent to maintain iron in solution. The initial concentration of each compound was set at 1 mg/L dissolved in a simulated effluent from a municipal wastewater treatment plant (MWTP). A factorial experimental design and its surface response analysis were used to optimize the operating parameters to achieve the highest initial degradation rate of each target. The evolution of the degradation process was measured by ultra-performance liquid chromatography (UPLC/UV), obtaining elimination rates above 90% for both contaminants. Statistical study showed the optimum concentrations of Fe(III) at 3 mg/L at an Fe-EDDS ratio of 1:2 and 2.75 mg/L H_2_O_2_ for the almost complete removal of the target compounds by solar photo-Fenton process. Validation of the experimental design was successfully carried out with actual MWTP effluent spiked with 100 μg/L of amoxicillin and acetaminophen, each at pilot plant scale.

## Introduction

The growing presence of microcontaminants in wastewater treatment plants (WWTPs), drinking water treatment plants (DWTPs), and even in the natural water bodies give an idea of the importance and great concern on what these emerging contaminants can potentially cause in the overall water cycle, as they may have medium- or long-term effects on both aquatic life and human health (Rogowska et al. 2020).

Among the microcontaminants that can be currently found in waters, pharmaceutical drugs have been the focus of research studies in the recent years, and both on account of their use and the risk, themselves or their degradation products, may suppose for the environment (Fatta-Kassinos et al. 2013; Patel et al. 2019; Krzeminski et al. 2019; Tran et a. 2018; Yang et al. 2020).

The production and consumption of pharmaceutical drugs determine the amount that may reach water bodies such as municipal wastewater treatment plants (MWTPs). A study by Choi et al. (2008) showed that the concentration of acetaminophen, carbamazepine, cimetidine, diltiazem, sulfamethoxazole, and trimethoprim in the environment followed the same order as the amount produced annually for each of these pharmaceuticals in Korea. High concentrations (above 10 g/L) of acetaminophen, tramadol, codeine, gabapentin, and atenolol were also detected in raw wastewater in Wales (UK), which could be explained by the high-prescribed amounts of these drugs (Kasprzyk-Hordern et al. 2009).

Although many types of drugs are produced worldwide, antibiotics and analgesics are the most important in terms of their volume of use due to the current high demand by consumers. In the case of antibiotics, there has been an increase of 36% over the last 10 years in 71 countries (Van Boeckel et al. 2014). Among the different types of antibiotics and analgesics, amoxicillin and acetaminophen are especially significant due to their high concentrations in the environment (Patel et al. 2019). Amoxicillin is a semi-synthetic antibiotic derived from penicillin. This aminopenicillin acts against a broad spectrum of bacteria, both Gram-positive and Gram-negative. Acetaminophen (paracetamol) is a drug with analgesic properties lacking clinically significant anti-inflammatory properties. It inhibits the synthesis of prostaglandins, i.e., the cellular mediators responsible for the onset of pain. It also has antipyretic effects (Zampronio et al. 2015).

The different pathways via which these contaminants can appear in wastewater range from human excretion, as some of them are not completely assimilated by the human body when used for medicinal purposes (European Commission, 215), to veterinary applications, in addition to uncontrolled discharges of medicines by pharmaceutical companies and from household surpluses due to a lack of environmental awareness (Kaczala and Blum, 2016).

There is clear evidence of their poor and inefficient removal in wastewater treatment plants using conventional technologies (Jelic et al. 2012; Petrovic et al. 2009; Petrie et al. 2015; Rizzo et al. 2019). Hence, the presence of these microcontaminants in natural waters comes from MWTP effluents. It is therefore essential to remove them from the main source of entry into ecosystems. This requires the application of tertiary treatments to eliminate these pollutants before they reach the environment. Advanced oxidation processes are among the treatments that have been demonstrated to be highly efficient in the abatement of wide variety of microcontaminants and pharmaceuticals (Affam et al. 2014; Velo-Gala et al. 2014).

Advanced oxidation processes (AOPs) comprise oxidative processes based on the generation of hydroxyl radicals (HO^**•**^), which are highly oxidizing species (E_0_=2.8 V with respect to the standard hydrogen electrode) and non-selective, highly desirable characteristics when degrading highly persistent microcontaminants (Wang and Zhuan 2020; Ribeiro et al. 2019).

There is, however, a factor to consider in all AOPs, namely the presence in natural waters of potential scavengers of these hydroxyl radicals, which significantly reduce the efficiency of the pollutant degradation process (Pignatello et al. 2006). Worth highlighting, in this respect, the presence of species such as CO_3_^2-^, HCO_3_^-^, SO_4_^2-^, Cl^-^ and humic acids. Within AOPs, Fenton process involves the reaction between iron ions and hydrogen peroxide to form reactive species capable of oxidizing different organic compounds. Additionally, photo-Fenton process could use sunlight as a source of radiation to increase the generation of HO^**•**^ and hence reaction efficiency. In the presence of UV or UV-visible radiation, photo-reduction of ferric ions in solution also occurs via a metal-ligand charge transfer reaction.

Studies in recent years on the application of solar photo-Fenton treatment to degrade low concentrations of microcontaminants in MWTP effluents have concluded that the operating conditions of the process do not have to be as aggressive as those for the treatment of industrial wastewater or effluents with a high organic load. In this regard, studies carried out by different authors (Clarizia et al. 2017; Zhan and Zhou 2019; Oller et al. 2021) have shown that high degradation rates can be obtained using low concentrations of iron and hydrogen peroxide and so reducing process operating costs.

The optimum pH for the photo-Fenton process is 2.8, because, under these conditions, the precipitation of hydroxides is avoided. This acidification in addition to the need for a neutralization step at the end of treatment, also supposes substantial treatment costs. Therefore, running the photo-Fenton process at near neutral pH could also mean cost savings depending on the iron complexing agent used (Silva et al. 2021; De Luca et al. 2014). Consequently, the study of the photochemistry of Fe(III) complexes plays a very important role (Souza et al. 2014; Ungwanen et al. 2020; Min et al. 2020) in the removal of microcontaminants when the aim is to determine the appropriate complexing agent that allows operating at neutral pH. Some of the Initial studies carried out in this field began working with ethylenediamine-N,N′-disuccinic acid (EDDS) (a structural isomer of Ethylenediaminetetraacetic Acid (EDTA)) as a complexing agent (Li et al. 2010; Wu et al. 2014). These studies showed that the complex of this compound with Fe^3+^ is stable in aqueous solution under neutral pH conditions and photochemically efficient. Three stereoisomers of EDDS exist, the most readily biodegradable stereoisomer being S, S‘]-EDDS (Schowanek et al. 1997; Metsarinne et al. 2001; Tandy et al. 2006; Huang et al. 2012; Lin et al. 2011).

There are several studies focused on the removal of microcontaminants at low concentrations in MWTP effluents using the photo-Fenton processes with complexes such as citric acid, oxalate, and EDDS. These include studies by (Li et al. 2010; Silva et al. 2007; Trovó et al. 2011; Huang et al. 2013; Klamerth et al. 2013) using EDDS as a complexing agent for the first time in MWTP effluents. At this point, it must be stressed that EDDS has been exhaustively studied by different authors up today (Ahmed et al. 2021; Soriano-Molina et al. 2021; Ahile et al. 2020). One of the key aspects when conducting these studies is the importance of the Fe(III):EDDS ratio, as it is crucial to determine the necessary ratio to obtain at least 90% degradation of the contaminants and carry out the photo-Fenton process at neutral pH. Klamerth et al. (2011, 2013) and Miralles-Cuevas et al. (2014) (Klamerth et al. 2013; Ahmed et al. 2021; Soriano-Molina et al. 2021; Ahile et al. 2020; Miralles-Cuevas et al. 2014) tested two different Fe(III):EDDS ratios, 1:1 and 1:2, concluding that an Fe(III) to EDDS ratio of 1:2 was slightly better than a ratio of 1:1 in terms of the degradation rate and hydrogen peroxide consumption.

The present work addresses the application and optimization of solar photo-Fenton oxidation process at neutral pH (by using EDDS) for the removal of two drugs: an antibiotic, amoxicillin and an analgesic, acetaminophen dissolved in simulated MWTP effluent for achieving degradation percentages higher than 90%. An experimental design has been carried out as an advancement regarding the state of the art in this topic, to optimize the concentration of reagents to maximize the initial microcontaminant degradation rate by solar photo-Fenton at neutral pH. In addition, optimized results have been applied to more actual operating conditions, changing the water matrix to an actual effluent of a MWWTP, to check the potential extrapolation of the mathematical model obtained.

## Materials and methods

### Reagents and chemicals

High purity (> 99 %) amoxicillin (AMX) and acetaminophen (ACT) were used (main characteristics are summarized in Table 1) for spiking simulated and actual MWTP effluents (physicochemical characterization shown in Table 2). Ethylenediamine disuccinic acid (EDDS 35% w/v) (as complexing agent) and catalase for removing H_2_O_2_ were supplied by Sigma-Aldrich, as well as solvents used for liquid chromatography analysis (99.9% grade). 75% purity ferrous sulphate monohydrate (Fe_2_ (SO_4_)_3_ H_2_O) and hydrogen peroxide H_2_O_2_ 35% (w/v) were supplied by Panreac.

**Table 1:**
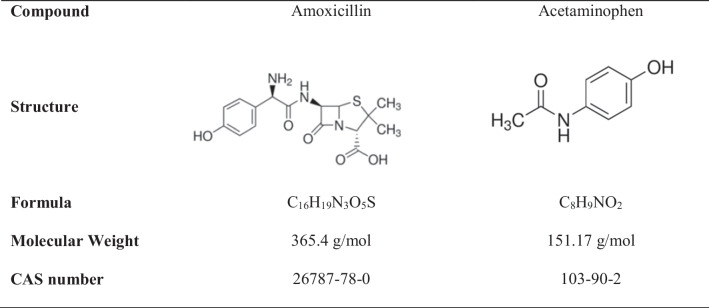
Main characteristics of amoxicillin and acetaminophen as target contaminants

**Table 2:** Physicochemical characterization of the simulated (obtained from a receipt of American Standard Methods) and actual MWTP effluent

	Simulated MWTP effluent	Actual MWTP effluent
pH	7.60	8.04
Conductivity (mS/cm)	1.50	2.31
Total organic carbon (mg/L)	14.8	35.0
Total inorganic carbon (mg/L)	13.5	155
Chloride (mg/L)	2.8	435.5
Ammonium (mg/L)	6.4	41.7
Potassium (mg/L)	14.8	26.1
Calcium (mg/L)	14.1	87.1
Nitrates (mg/L)	23.2	4.5
Magnesium (mg/L)	11.3	69.7
Sulphates (mg/L)	96.0	129.7
Sodium (mg/L)	24.6	274.1

### Analytical procedures

Target contaminants at low concentrations were measured by ultra-performance liquid chromatography (UPLC-UV) (Agilent Technologies, Series 1200) employing a Gemini C-18 column. Starting conditions were 100% 10 mM KH_2_PO_4_ buffer (mobile phase A) and 0% ACN (mobile phase B). A linear ramp was applied with the following sequence: 40% (phase A) to 60% (phase B) in 10 min and 0% (phase A) to 100% (phase B) in 13 min. The detection wavelengths and the limits of quantification (LOQ) and detection (LOD) for each contaminant are shown in Table 3.

**Table 3:** Detection wavelengths, limits of quantification (LOQ), and limits of detection (LOD) for each contaminant in UPLC-UV

	AMX	ACT
Wavelength (nm)	230	245
Correlation	0.9994	0.9999
Slope	121.6	358.6
Intercept	1.6	4.3
Intercept 0	123.8	364.7
LOD	0.0040	0.0014
LOQ	0.0120	0.0041

Dissolved organic carbon (DOC) and inorganic carbon (IC) were determined on a Shimadzu TOC-VCSN analyser. The samples were filtered through 0.22 μm before analysis so that only the dissolved organic carbon (DOC) was determined. DOC analysis was always performed immediately after collecting the sample.

Total iron concentration was measured using the 1,10-phenanthroline method according to ISO 6332. Hydrogen peroxide was measured using titanium oxysulphate (IV) following DIN 38402H15.

### Experimental procedure and design

A series of preliminary experiments were performed to determine the level of each factor mostly affecting the degradation of the contaminants. A mixture of 1 mg/L of each amoxicillin and acetaminophen dissolved in simulated MWTP effluent was used in these trials.

**Fig. 1:**
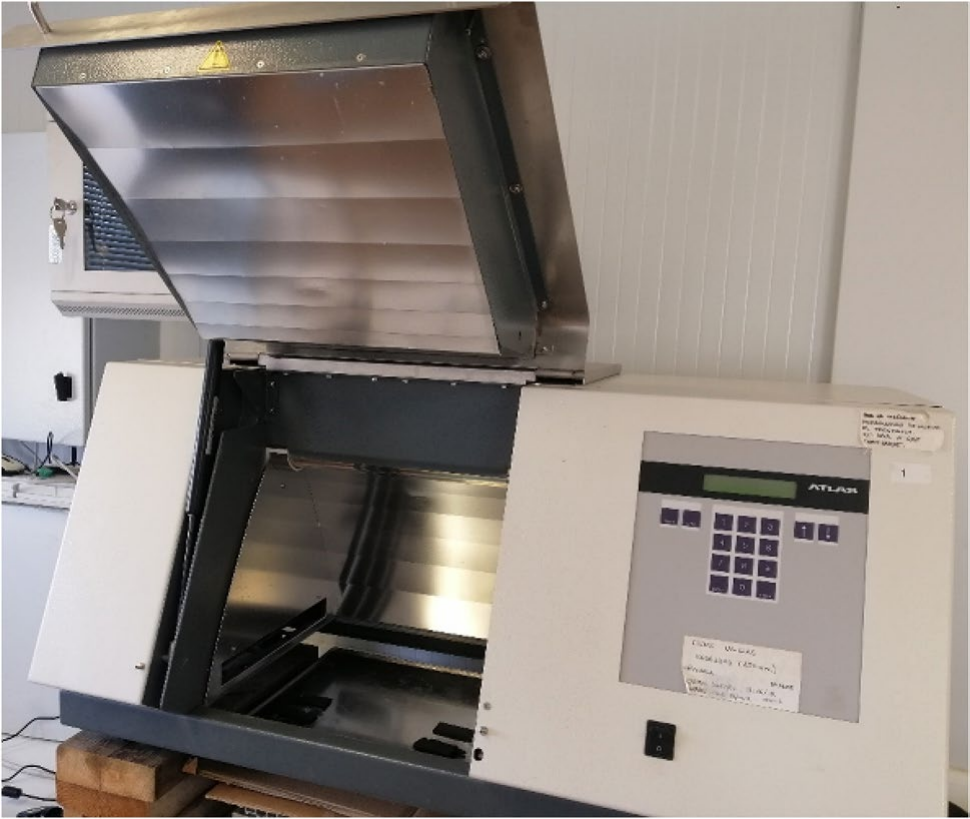
Experimental device employed in the performance of the experimental design, Suntest, XLS + Heraeus, Germany

The experiments were run using a solar simulator (Suntest, XLS + Heraeus, Germany) equipped with a 2.2 kW xenon arc lamp and a special glass filter (daylight) that cuts off UV irradiation at 290 nm. The radiation from the lamp was measured using a pyranometer (Solar Light PM A2100) (Fig. 1).

Initially, a total volume of 1.5 L of simulated MWTP effluent was spiked with 1 mg/L of each pharmaceutical and then stirred for several minutes to ensure a suitable degree of homogeneity. Subsequently, the concentration of Fe^3+^-EDDS was added to the mixture, and an initial sample was taken to ensure that complex formation had taken place and for checking the pH. The reason for using Fe_2_ (SO_4_)_3_ H_2_O as a source of Fe^3+^ in this paper is that previous studies showed that the iron source can strongly influence the degradation of pollutants^44^, in addition to the possibility of working with the Fe^3+^-EDDS complex at neutral pH and minimizing operating expenses, as regent costs are thus reduced considerably.

The complex was previously prepared in the laboratory immediately before used by dissolving the required iron quantity (depending on the concentration to be tested) in demineralized water at pH 3 and adding the corresponding EDDS amount in the dark, as the complex is affected by light. Then, the mixture was left to homogenize for 5–10 min, and the solution turned bright yellow in colour, indicative of the correct formation of the complex.

After, required hydrogen peroxide concentration (depending on the experiment) was added, and the photo-Fenton reaction began. The total reaction time was 30 min, taking samples every 2 min. Catalase was used to stop the reaction. This enzyme is able to neutralize oxygen-derived forms (such as H_2_O_2_). The amount of catalase was added as function of the initial concentration of H_2_O_2_ employed.

Experiments were performed in a borosilicate glass beaker under constant stirring. The beaker was 19 cm diameter, providing an irradiated surface of 0.0284 m^2^, and the entire volume was illuminated 1.5 L.

The variables studied to degrade 90% of the contaminants in the preliminary experiments were the treatment time, initial degradation rate, accumulated UV energy (*Quv*), Fe^3+^-EDDS (1:2) complex concentration, H_2_O_2_ concentration, and dissolved organic carbon (DOC). The pH was around 7 throughout the reaction. *Q*_*uv*_ was calculated according to Eq 1:

Eq. 1 $${Q}_{uv}\left( kJ/l\right)=\sum_n\overline{\textrm{U}{\textrm{V}}_{n-1}}\left(W/{m}^2\right)x\frac{A_r\left({m}^2\right)}{V_{\textrm{total}}(L)}x\frac{{\varDelta t}_{n-1}\left(\min \right)}{1000}$$

where *Q*_UV_, is the UV energy accumulated per litre (J/L) at times *n*; (UV) ®*n*-1 is the average incident radiation on the irradiated area (W/m^2^); Δ*t*_*n*-1_ is the experimental time of the sample; *A*_*r*_ is the illuminated area (m^2^); and *V*_total_ is the total volume (L).

Once the influencing range of each target parameter (Fe^3+^:EDDS and H_2_O_2_ concentrations) had been determined, a central composite design was selected as a statistical and mathematical method normally used to select the best experimental conditions requiring the lowest number of experiments (Sarrai et al. 2016; Idil et al. 2009). In this case, the design has involved a set of 14 experiments to find the optimum values of these variables for the maximum degradation of the drugs with the minimum accumulated UV energy required. This design includes low (−) and high (+) levels and center points for Fe^3+^:EDDS and H_2_O_2_ concentrations. It is based on a star design with two variables at two levels, consisting of three types of experiments: 2 *k* coded values (in our case *k* = 2 and consists of four experiments), four modified axial or star points (± 1.01 and 0 instead of normalized ± 1.41 in order to avoid negative concentration values), and three replicates of the central point (0,0). The Minitab15® software statistical tool was employed to analyse the central composite design and to plot the response surfaces. The response factors considered in this study were the initial degradation rate of each target contaminant. Optimum concentrations of both reagents H_2_O_2_ and Fe^3+^:EDDS to maximize both initial degradation rates with the minimum accumulated UV energy required were also determined. Both reagents’ concentrations are considered critical points in the optimization and economic evaluation of a photo-Fenton degradation process.

The experimental procedure followed in these tests was the same as for the previously described assays.

After completing the experimental design, two tests were developed with the main goal of validating the values of each parameter defined as optimal to maximize the initial degradation rate for each contaminant. Two different aqueous matrices were used in those validation experiments, simulated water from the MWTP (see Table 2) and actual effluent from El Ejido MWTP (south-east of Spain), to which the content of HCO_3_^−^/CO_3_^2−^ (hydroxyl radical scavengers^21^) was eliminated by the addition of concentrated H_2_SO_4_ just in a sufficient amount to avoid a significant drop in pH. In such experiments, a photoreactor pilot plant based on CPC (compound parabolic collectors) was used at the Plataforma Solar de Almería (PSA, latitude 37° N, length 2.4 W) using natural solar radiation. The initial concentration of drugs, AMX, and ACT was 1 mg/L each. After 12 min of homogenization of the aqueous matrix together with the drugs in the CPC photoreactor, an initial sample was taken to ensure the starting concentration of both contaminants. The Fe^3+^ -EDDS complex was then added leaving a time for homogenization, and finally, the first dose of hydrogen peroxide was added. The photoreactor was then uncovered, and the solar photo-Fenton reaction started. Samples were taken every 3 min during the first hour in the simulated effluent from the MWTP and every 5 min for the first 60 min of reaction in the actual MWTP effluent assay and thereafter every 15 min to complete 3 h of treatment.

## Results and discussion

A series of preliminary studies were conducted with the aim of selecting the best operation range of influencing parameters for the experimental design, stablishing upper concentration limits on 5.5 mg/L Fe^3+^ and 5 mg/L H_2_O_2_ (added as consumed) according to previous published works (Klamerth et al. 2013; Ahmed et al. 2021; Soriano-Molina et al. 2021; Ahile et al. 2020; Miralles-Cuevas et al. 2014; Nogueira et al. 2005; Prieto-Rodríguez et al. 2013). This concentration of H_2_O_2_ was chosen because previous studies by Huang (Huang et al. 2013) showed that higher concentrations involve loss of the positive effect of the addition of EDDS over the process, because hydrogen peroxide in excess also acts as a scavenger of hydroxyl radicals, thereby decreasing the efficiency of the system. The results of these preliminary tests gave high degradation rates of both compounds (above 90%) in only 9 min of exposure.

Based on these results and previous published studies (Prieto-Rodríguez et al. 2013; Klamerth et al. 2011) which showed that high degradation rates can be obtained using low concentrations of iron and hydrogen peroxide, four tests were carried out setting the concentration of Fe^3+^ at 5.5 mg/L and doing additions of 5 mg/L of H_2_O_2_ as consumed.

First, it should be noted that the concentration of Fe^3+^ was almost constant throughout the reaction time so Fe-EDDS complex was stable along the whole process. More than 94% of both contaminant degradation was attained after only 3.6 mg/L of H_2_O_2_ consumed and 6 min of reaction time.

According to these previous results, the range of influencing target parameters selected for the experimental design was 0.5–5.5 mg/L for Fe^3+^-EDDS complex and 2.75–5 mg/L for H_2_O_2_ with the aim of maximizing the initial degradation rate of both target contaminants with the minimum accumulated UV energy required.

### Key parameter optimization

Once the range of each key parameter had been selected, a central composite design was applied with the aim of optimizing the operational parameters to maximize pharmaceutical degradation rate through a solar photo-Fenton process at neutral pH. The experimental design established the necessity to perform 14 experiments in which both drug initial concentration was stated at 1 mg/L. The pH remained constant in all trials between 7 and 8. The wastewater matrix used for tests at laboratory scale was simulated effluent from a MWTP (characterization shown in Table 2).

Irradiation was set at 30 W/m^2^ in the solar box according to the irradiation obtained in the mid-day of a sunny day. Both the irradiated area and the reactor volume were kept constant, so *Quv* was only the function of the sampling time interval, reaching values of 1.02 (kJ/L) at the end of each test (30 min). In consequence, in trials using the maximum concentrations of Fe^3+^ and H_2_O_2_ (5.5 mg/L and 5 mg/L, respectively), drug degradation rates of 50% were achieved without irradiation that is thanks to Fenton reaction. However, *Quv* values of 0.75 kJ/L were required to obtain this same degradation percentage in trials employing low concentrations of Fe^3+^ and H_2_O_2_. Table 4 shows the experimental conditions and results obtained in the 14 experiments designed. It also shows the degradation rates of both compounds obtained by representing the evolution of the concentration of the drugs versus time corresponding to kinetics of order 1.

**Table 4:** Central composite design matrix for lab scale experiments including target contaminant degradation percentages and their corresponding initial degradation rates

Experiments	Fe(III) mg/L	H_2_O_2_ mg/L	Degradation % AMX	Degradation % ACT	Initial degradation rate AMX (mg/L min)	Initial degradation rate ACT (mg/L min)
1	0.5	0.5	76	48	0.184	0.108
2	5.5	0.5	66	61	0.143	0.125
3	0.5	5.0	70	70	0.205	0.126
4	5.5	5.0	96	91	0.300	0.289
5	3.0	2.75	93	91	0.307	0.305
6	3.0	2.75	95	94	0.290	0.276
7	3.0	2.75	91	87	0.308	0.324
8	0.5	0.5	71	35	0.173	0.107
9	5.5	0.5	68	61	0.119	0.134
10	0.5	5.0	70	60	0.244	0.196
11	5.5	5.0	98	98	0.350	0.352
12	3.0	2.75	92	90	0.308	0.319
13	3.0	2.75	93	92	0.294	0.305
14	3.0	2.75	94	93	0.306	0.326

It can be seen that the higher the concentrations of Fe^3+^-EDDS and H_2_O_2_ (experiments 4 and 11), the greater the degradation percentages obtained, which corresponds with a higher initial degradation rates for both contaminants. Values close to those reported above are also achieved for intermediate values of Fe^3+^-EDDS and H_2_O_2_ concentrations (3 mg/L and 2.75 mg/L, experiments 5, 6, 7, 12, 13, and 14). Although 100% removal was not achieved in any of these cases, even when increasing the concentration of iron and hydrogen peroxide, high primary degradation of contaminants did occur. These degradation results are consistent with those reported by Trovó et al. (2008), who studied the degradation of amoxicillin (AMX), paracetamol or acetaminophen (ACT), and bezafibrate (BZF). Working with concentrations of 1.0 to 5.0 mM H_2_O_2_ and using potassium ferrioxalate (K_3_Fe (C_2_O_4_)_3_ 3H_2_O) or Fe(NO_3_)_3_ as a source of Fe^3+^ at optimum pH for the photo-Fenton reaction (2.8), these authors found that degradation is favoured by (K_3_Fe (C_2_O_4_)_3_·3H_2_O), achieving 98% removal of BZF and ACT after 5 min of irradiation. However, no difference was observed in the oxidation of AMX, for which complete degradation was achieved after 0.5 min of irradiation time for both iron sources. Nonetheless, it should be noted that the pH employed in the present study was close to neutral, while Trovó et al. studies were carried out at optimal pH of 2.8 for photo-Fenton applications to avoid iron precipitation.

The DOC measurement showed that the value remained practically constant throughout the experiments, thus demonstrating that the complex was still active and the EDDS was not yet degrading.

Initial degradation rate for each target contaminant is considered in the proposed experimental design as the response factor.

Based on these results, an analysis of variance was carried out using the statistical program MINITAB to study how the different process variables and the interaction between them affected the percentage degradation of the contaminants. Although both variables influence the process, presenting a *p*-value < 0.05, Fe^3+^ affects the photo-Fenton process less than H_2_O_2_, as evidenced by the values of the effects and the Student-*t* values shown in Table 5.

**Table 5:** Summary of the variables studied in the central composite design and the statistical values obtained

Variables	Effects	Student-*t*	*p*-value
Fe(III)	0.1214	3.43	0.009
H_2_O_2_	0.2384	6.73	0.000
Fe(III)*H_2_O_2_	0.1606	4.54	0.002
Central point		8.18	0.002

A statistical study carried out by MINITAB software gave different graphics allowing the analysis of the influence rate of each parameter on the initial degradation rate for each target drug.

The rate of degradation of these compounds under the experimental conditions in which the experiments were carried out is a first-order reaction:

$$-\textrm{d}\left[\textrm{Contaminants}\right]/\textrm{dt}=\textrm{k}\ast \left[\textrm{Contaminants}\right]$$

The optimization of both contaminant degradation by solar photo-Fenton process accordingly to the selected experimental design gave the following common initial degradation rate (*r*_0_) equation (Eq. 2):

Eq. 2 $${r}_0=0.2846\hbox{--} 0.0150\ {\textrm{Fe}}^{3+}+0.0101\ {\textrm{H}}_2{\textrm{O}}_2+0.01428{\textrm{Fe}}^{3+}{\textrm{H}}_2{\textrm{O}}_2\left({R}^2=94\%\right)$$

The statistical study allowed obtaining the equations, which model the initial degradation rate for each contaminant as function of the concentrations of the influence parameters (Fe-EDDS and H_2_O_2_) and for the sum of both. Such equations are shown in Table 6. High accuracy of the proposed statistic models is demonstrated thanks to *R*^2^ values between 94 and 96%. These results coincide with those obtained by other authors (Rodriguez et al. 2005).

**Table 6:** Initial degradation rate predictive model equations for each target contaminant given by the statistic program according to the experimental design results

	Predictive model equation	*R*^2^
Degradation rate AMX	*r*_0_ = 0.413 + 0.071 Fe(III) + 0.007 H_2_O_2_ – 0.014 Fe(III)*Fe(III) + 0.007 Fe(III)*H_2_O_2_	96% (Eq. 3)
Degradation rate ACT	*r*_0_ = 0.044 + 0.126 Fe(III) + 0,009 H_2_O_2_ – 0.021 Fe(III)*Fe(III) + 0.006 Fe(III)* H_2_O_2_	94% (Eq. 4)

**Fig. 2:**
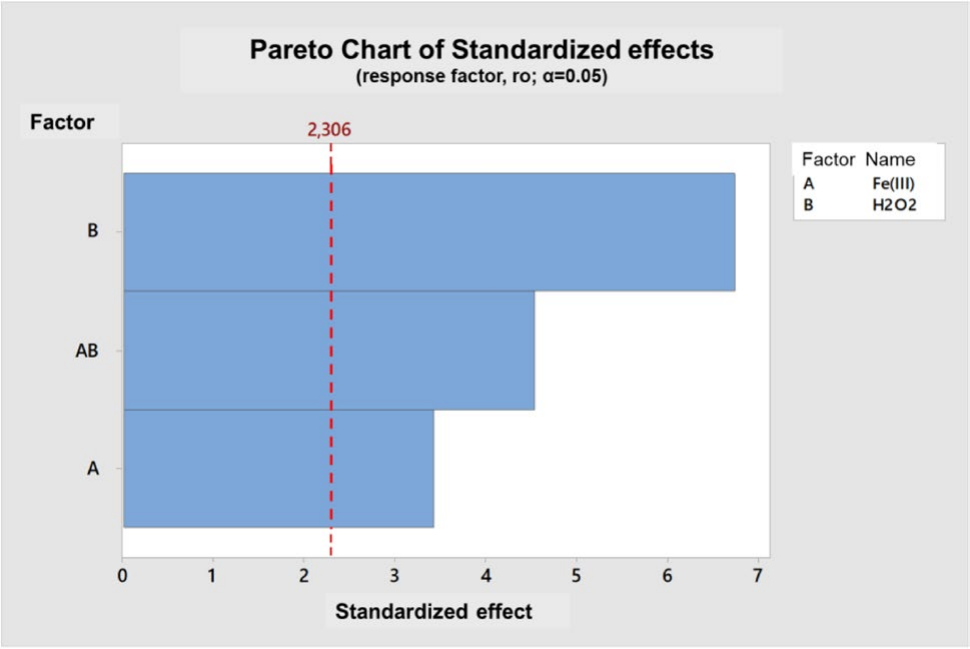
Pareto chart showing the influence of each variable (Fe(III) in the EDDS complex and H_2_O_2_) versus a standardized effect

The Pareto chart (Fig. 2) was used to decide which of the variables or the interaction between them was more significant. This type of chart shows both the magnitude and the significance of the effects. The vertical axis represents the two variables and the interaction between both, while the horizontal axis represents the standardized effects. In this case, the effects of the variables are specified in the model equation and show the difference between the maximum and minimum for each value. The vertical reference line indicates that any effect that extends beyond this line is significant. The Pareto chart for this study confirms that the most important variable is the concentration of hydrogen peroxide and the interaction of this reagent with Fe.

**Fig. 3:**
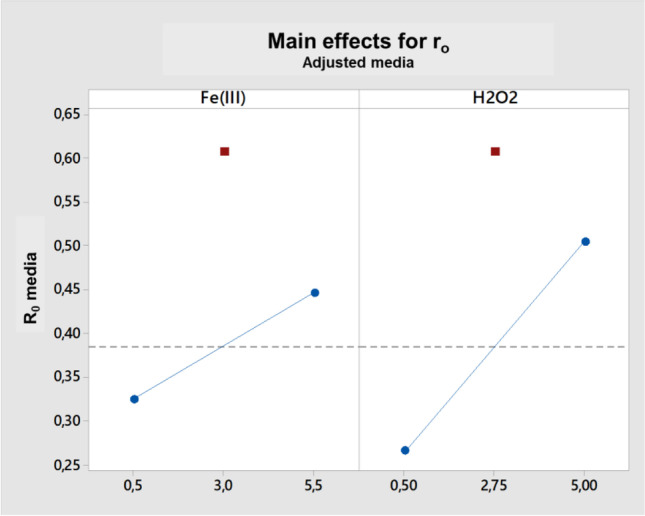
Main effect graph of each selected variable (Fe (III) complexed with EDDS and H_2_O_2_) on the initial degradation rate for each target contaminant (response factor)

Figure 3 shows the individual effects of each variable (Fe^3+^ and H_2_O_2_) on the initial degradation rate of the target contaminants. It can be seen that the influence of both parameters is positive as their concentration is increasing (blue line in Fig. 3). That means that no limitation was observed, and, initially, higher initial degradation rates for both contaminants should be attained as increasing both Fe^3+^ and H_2_O_2_ concentrations (red dots in Fig. 3 indicate the central point of the experimental design).

**Fig. 4:**
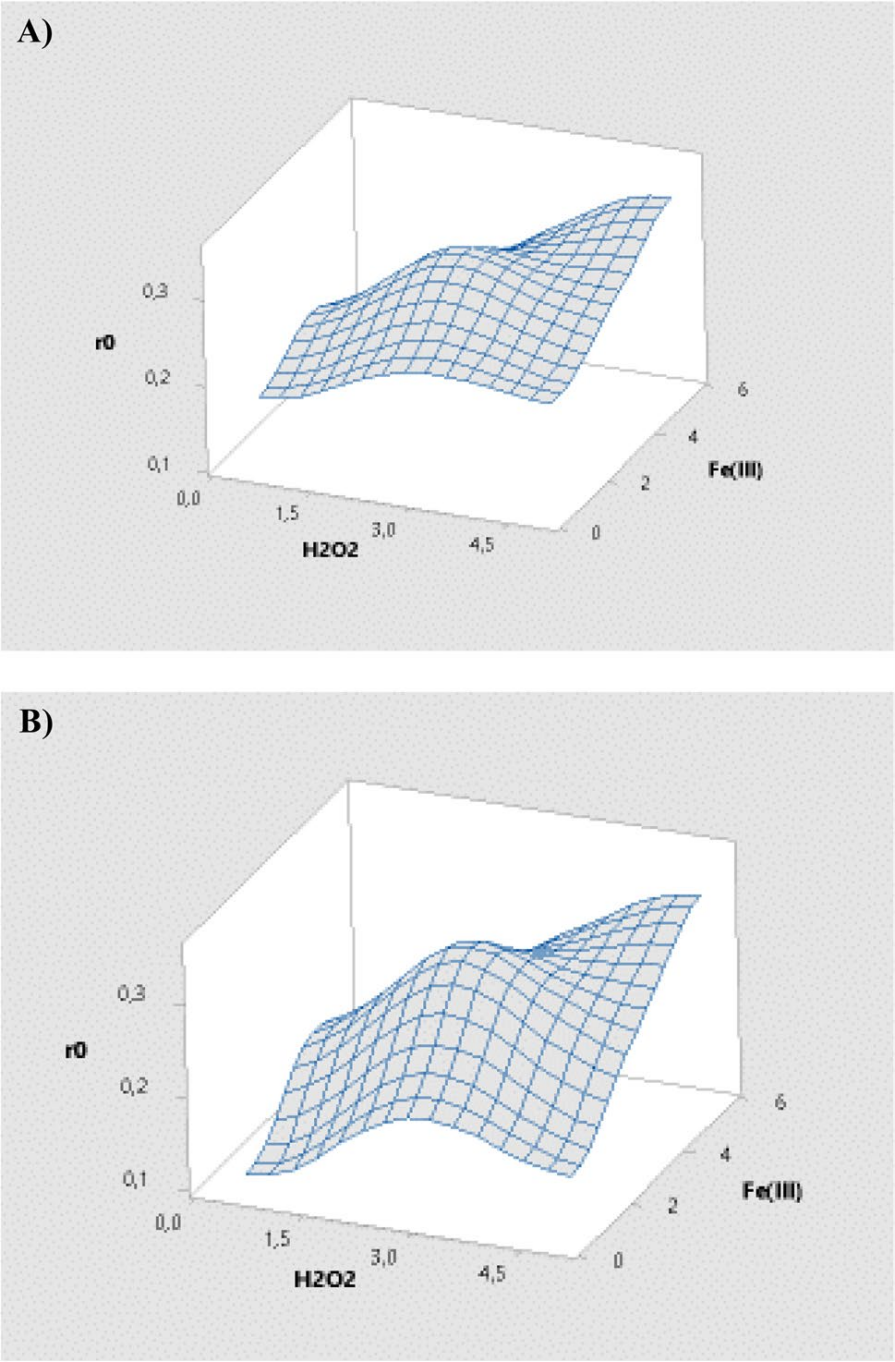
Response surfaces showing the variation of influence of each variable (Fe (III) complexed with EDDS and H_2_O_2_) on the initial degradation rate of **A** AMX and **B** ACT

The response surface graphics have been obtained in the experimental design (Fig. 4). It must highlight the maximum response observed in the central points (at 3 mg/L of Fe^3+^ and 2.75 mg/L of H_2_O_2_) similar to that shown at the maximum values of both parameters. As maximum degradation rates were not significantly improved at the maximum concentration of both parameters, it was decided to choose the central point values as the optimal operation conditions for the degradation of such contaminants. Indeed, this is what it was obtained in the system optimization represented by Fig. 3.

**Fig. 5:**
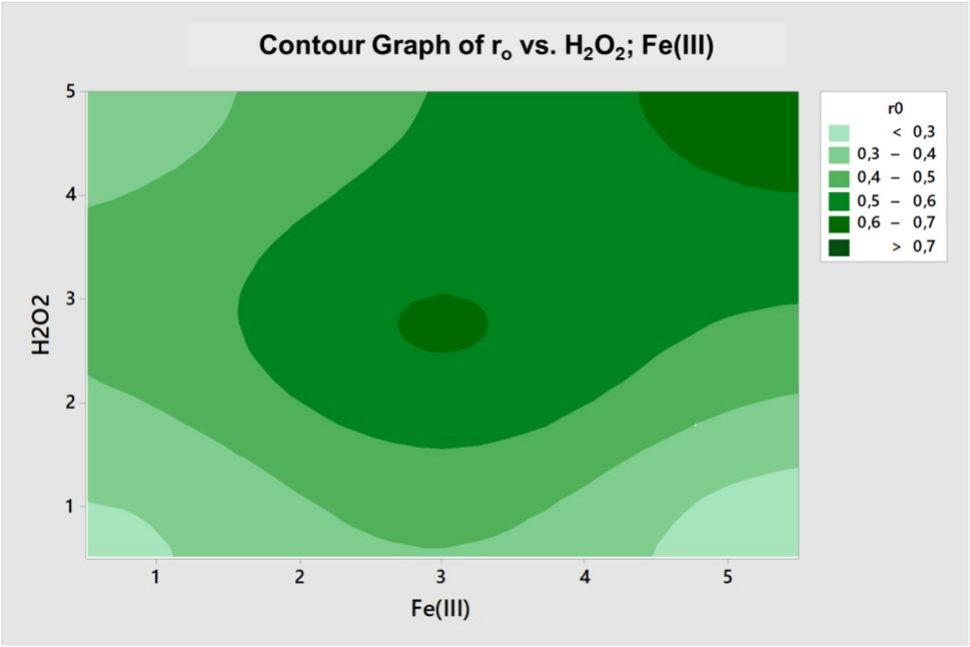
Contour graph showing the influence potential of each studied range for each variable (Fe(III) complexed with EDDS and H_2_O_2_) on the initial degradation rate of both target contaminants

It is clear from the contour plot (Fig. 5) that, although the optimal degradation value is also obtained in the experiment with the highest concentration of Fe and H_2_O_2_, the maximum degradation rate actually appears at the central points (darker area in the figure). Given that 90% degradation rates were obtained using the reagent concentrations corresponding to these central points within the same reaction time as that achieved when using the maximum reagent concentrations, as previously mentioned, it was decided to consider these central point as optimal to validate the predictive model equations mathematically obtained, as reagent costs would be minimized.

**Fig. 6:**
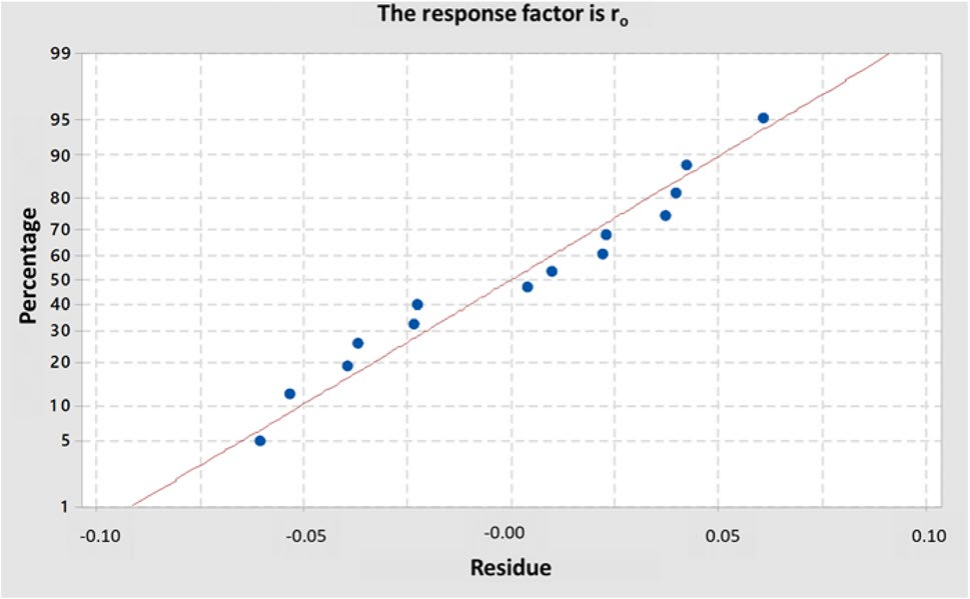
Normal probability plot of the residuals from the statistical model obtained in the central composite design

Residual curves from the obtained statistical model have been also evaluated. As it is recommended, to evaluate residue normality, the normal probability plot of the residuals is presented in Fig. 6. As it can be observed, it shows the residuals vs. their expected values when the distribution is normal.

Normally, the probability plot of the residuals is used to verify the assumption that the residuals are normally distributed. The normal probability plot of the residuals should follow approximately a straight line as it occurs in Fig. 6. In addition, there are any points far enough from the line which could imply a distribution with outliers.

### Application of the optimized reagent concentration to actual operating conditions

Once the optimum concentration values for the Fe^3+^-EDDS complex and H_2_O_2_ were selected according to the experimental design results, two more experiments were carried out at pilot plant scale to check the reliability of the model under more realistic operating conditions. First, the elimination of both drugs spiked at 1 mg/L each one (same concentration as those initially used for the experimental design) in actual MWTP effluent (see Table 2 for physic-chemical characterization) was performed. The initial degradation rates obtained were 0.021 mg/L min for AMX and 0.015 mg/L min for ACT, respectively, attaining global degradation percentages of 47% for AMX and 20% for ACT, respectiverly, after 5 min of irradiation time. After the first 5 min of treatment, three extra additions of H_2_O_2_ (5 mg/L each) were made in order to determine the maximum percentage of degradation that could be reached for both drugs, which was around 60% for both after 180 min of treatment and a total H_2_O_2_ consumption of 13.4 mg/L. pH remained constant throughout the reaction between 7.6 and 7.9. DOC remained also constant along the 180 min. Iron concentration decreased till around 1.7 mg/L at the end of the treatment.

These results showed that the greater physic-chemical complexity of the actual effluent from the MWTP caused a reduction in the efficiency of the treatment and the necessity of a greater consumption of H_2_O_2_ and higher treatment times to attain quite lower degradation rates than those obtained with simulated effluent from the MWTP (Fig. 6). Specifically after 5 min of solar photo-Fenton treatment at neutral pH, 90% of degradation was obtained for both drugs with an initial degradation rate around 0.3 mg/L min (15 times higher) in simulated MWTP effluent (Table 4).

Finally and with the main objective of checking the performance of solar photo-Fenton optimum parameters at pilot scale (3 mg/L Fe^3+^ (1:2 Fe: EDDS) and 2.75 mg/L H_2_O_2_) and under more realistic conditions considering lower target contaminant concentration, experiment with actual effluent of MWTP was repeated by spiking 100 μg/L of ACT and AMX, each. Ninety-one percent of AMX degradation was achieved after 105 min of treatment and 11 mg/L of H_2_O_2_ consumption. However, at that time, only 17% of ACT was eliminated, so solar photo-Fenton process was maintained till achieving 70% of ACT degradation after 210 min and 13.1 mg/L of H_2_O_2_ consumption. As already commented, the more complex water matrix provoked the requirement of higher doses of H_2_O_2_ to achieved contaminant degradation targets, that was, five additions of 2.75 mg/L of H_2_O_2_ for maintaining the reaction till maximum ACT elimination (most unfavorable condition).

## Conclusions

The use of the Fe(III)-EDDS complex has been widely studied and is a very well-known strategy to apply solar photo-Fenton at neutral pH to eliminate microcontaminants contained in secondary effluents of municipal wastewater treatment plants. However, optimization of the low concentration of reagents and the elaboration of mathematical/theoretical models that could allow making reliable extrapolations to more actual conditions is not available, yet. In this work, Fe(III) concentration complexed with EDDS and H_2_O_2_ concentration have been optimized to 3 mg/L for Fe^3+^ and 2.75 mg/L for H_2_O_2_ according to a central composite design for the maximization of the initial degradation rate of two target microcontaminants. In addition, mathematical model equations have been obtained for predicting the behaviour of the system and trying to calculate the response factor under more actual operating conditions.

The use of the optimum concentrations of the variables, obtained in the experimental design, for the treatment of actual effluent spiked with the same concentration of target pollutants showed lower initial degradation rates than those expected. The consumption of H_2_O_2_ was always higher than the optimum to reach the same point obtained with simulated wastewater due to the presence of other substances that consume hydroxyl radicals, such as organic matter as humic acids, carbonates, and bicarbonates.

It was found that, when operating at the aforementioned reagent concentrations using solar photo-Fenton at neutral pH, primary degradation rates of 90% and 70% of target contaminants under more realistic operating conditions (100 μg/L, each, spiked in real MWTP effluent) were achieved, though higher final doses of H_2_O_2_ and longer treatment times were required. These results confirm some previous studies that suggest the necessity of applying optimization statistic tools under the most actual operating conditions as possible to be able to predict deviations in the system accurately. Nevertheless, as shown in this work, small changes in the physicochemical characterization of the actual wastewater could lead to a lack of confidence in the model provoking the necessity of performing additional confirming experiments.

## Data Availability

We ensure that all data and materials as well as software application or custom code support their published claims and comply with field standards.
